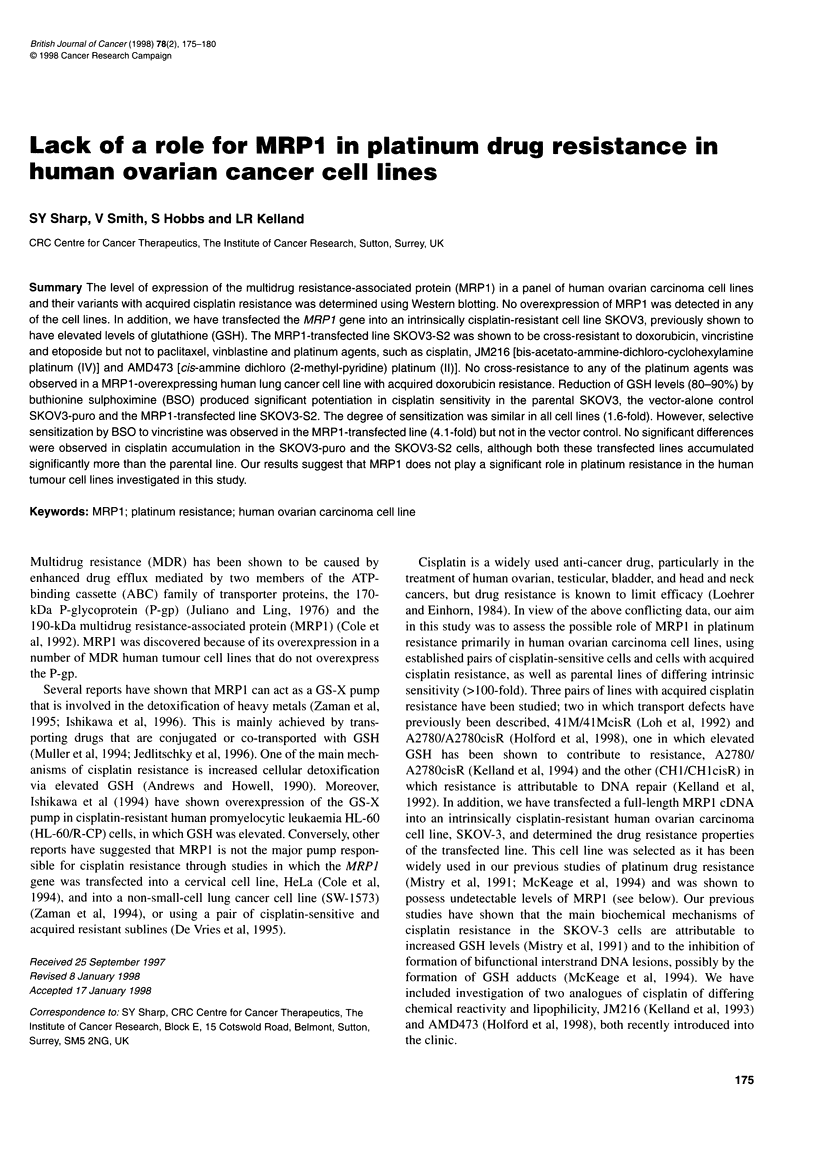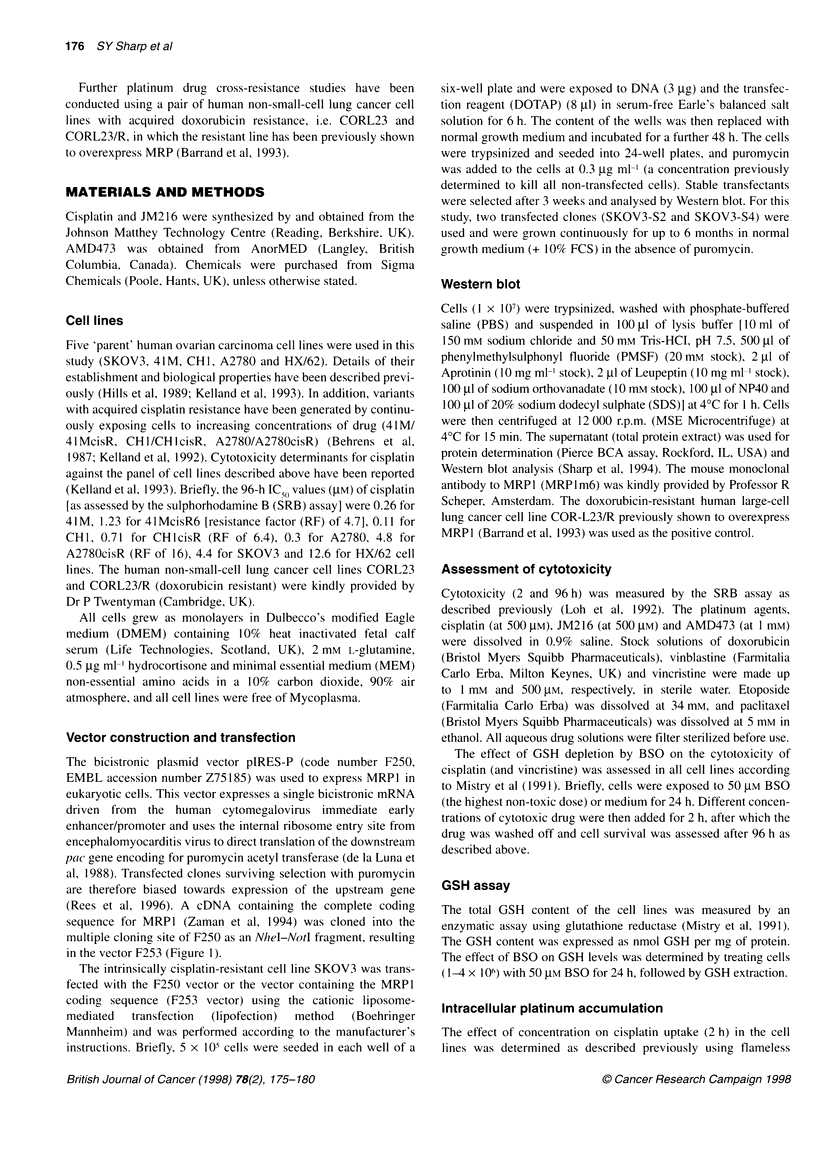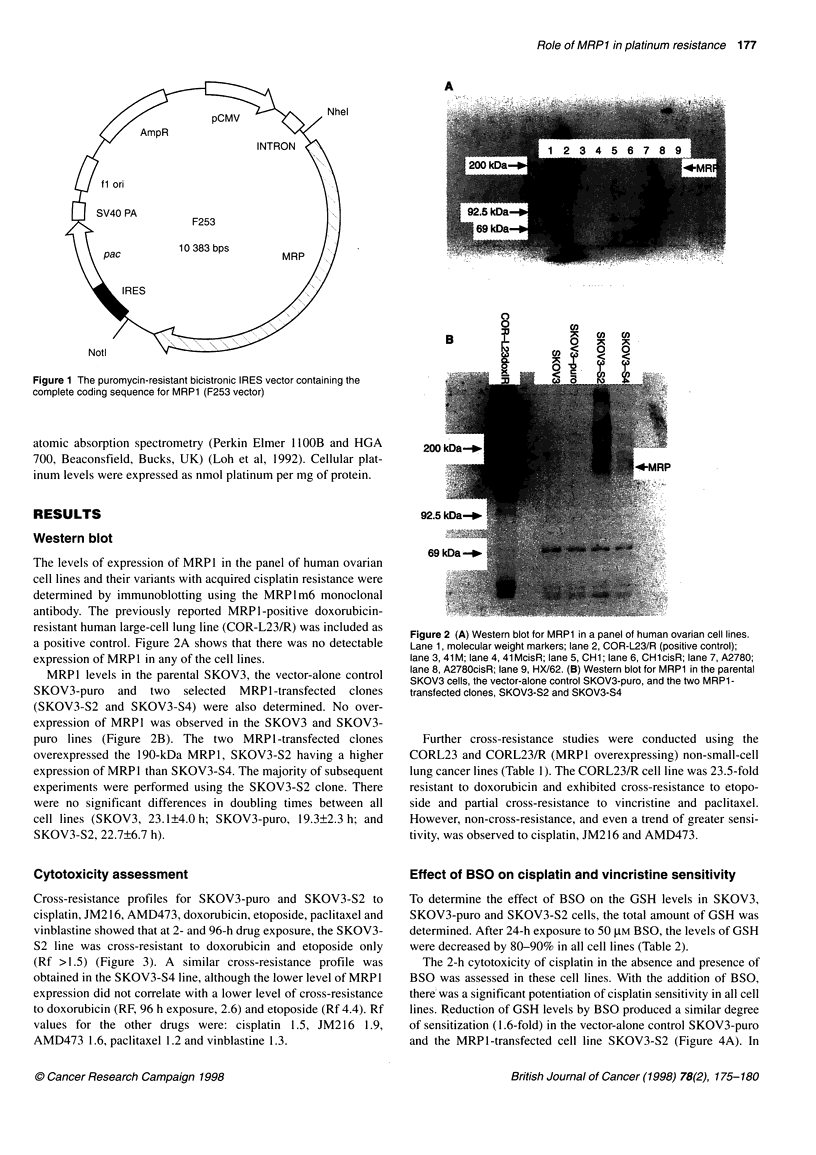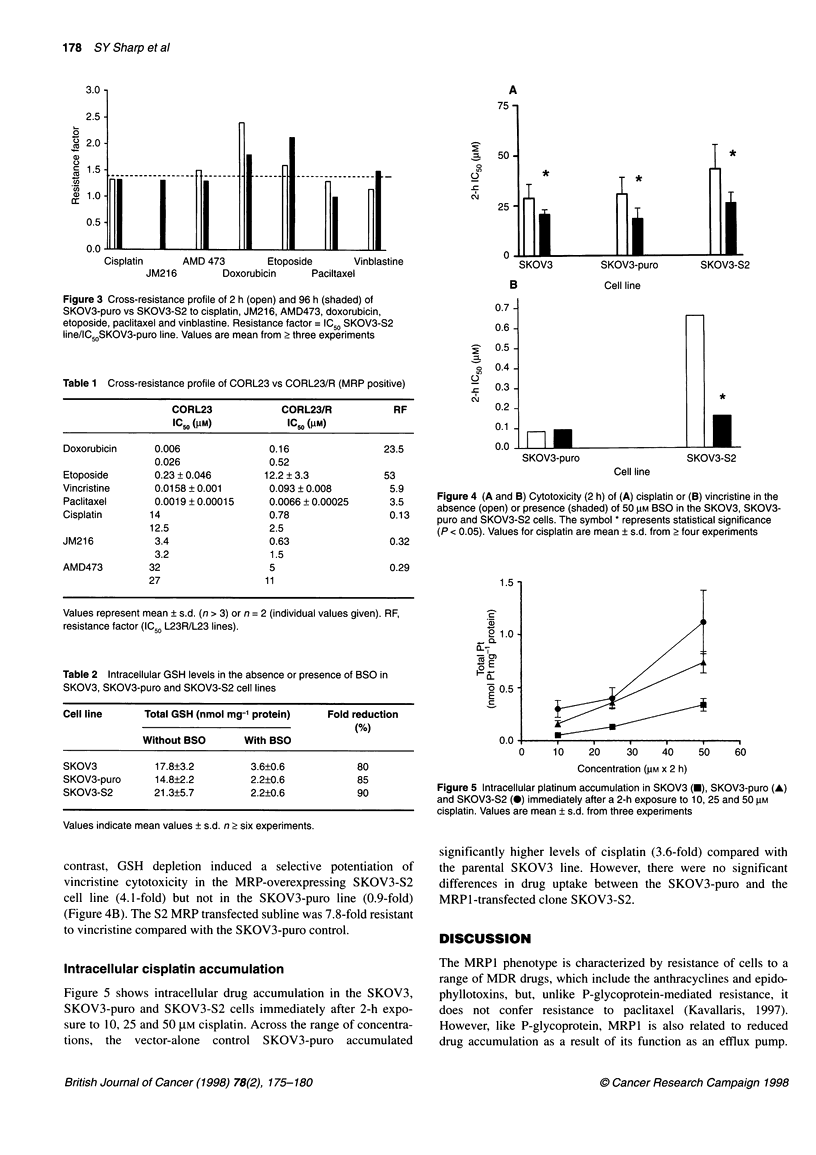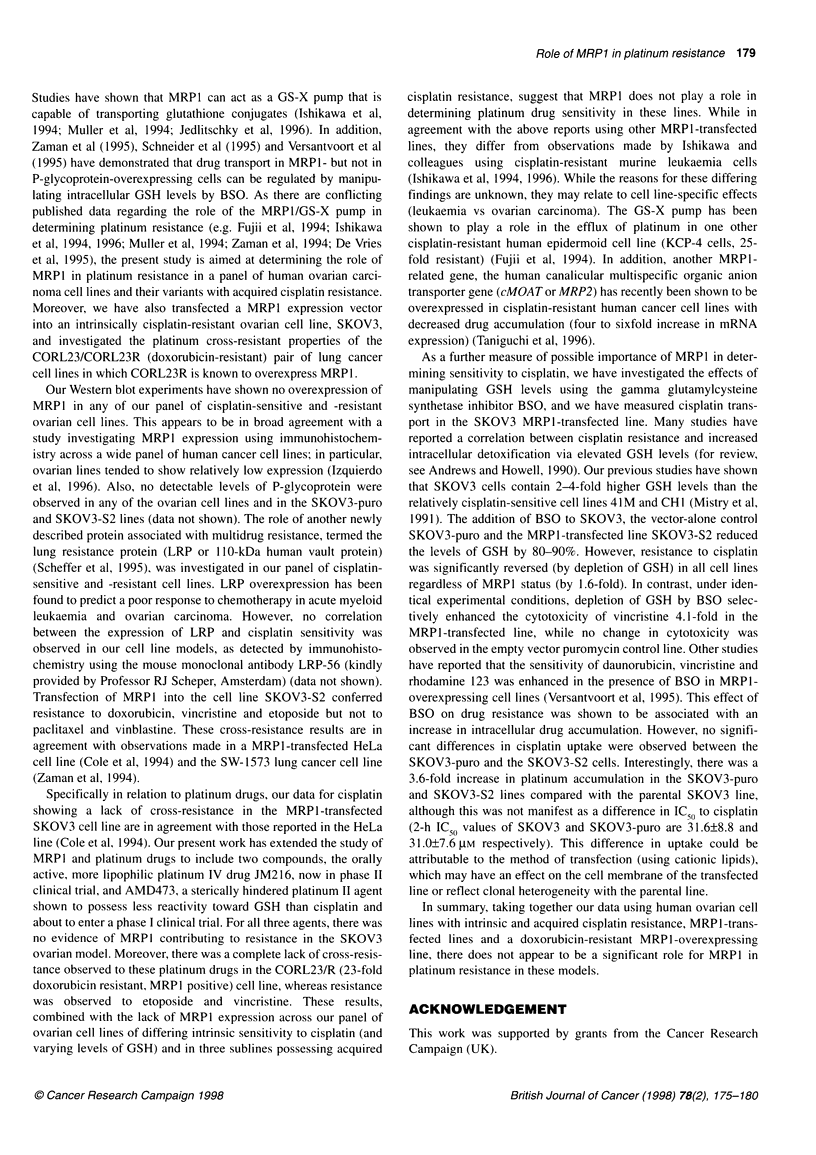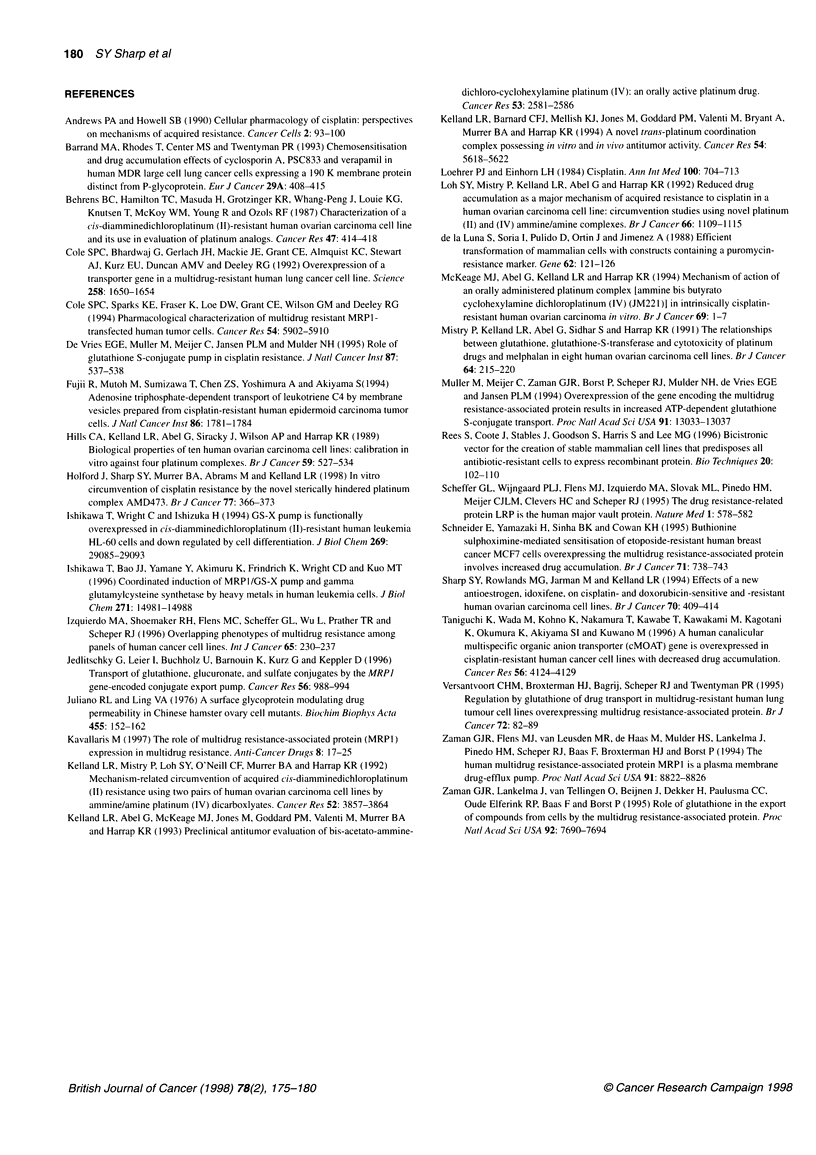# Lack of a role for MRP1 in platinum drug resistance in human ovarian cancer cell lines.

**DOI:** 10.1038/bjc.1998.461

**Published:** 1998-07

**Authors:** S. Y. Sharp, V. Smith, S. Hobbs, L. R. Kelland

**Affiliations:** CRC Centre for Cancer Therapeutics, The Institute of Cancer Research, Sutton, Surrey, UK.

## Abstract

**Images:**


British Journal of Cancer (1998) 78(2), 175–180
© 1998 Cancer Research Campaign

### Lack of a role for MRP1 in platinum drug resistance in human ovarian cancer cell lines

**SY Sharp, V Smith, S Hobbs and LR Kelland**

CRC Centre for Cancer Therapeutics, The Institute of Cancer Research, Sutton, Surrey, UK

**Summary** The level of expression of the multidrug resistance-associated protein (MRP1) in a panel of human ovarian carcinoma cell lines and their variants with acquired cisplatin resistance was determined using Western blotting. No overexpression of MRP1 was detected in any of the cell lines. In addition, we have transfected the *MRP1* gene into an intrinsically cisplatin-resistant cell line SKOV3, previously shown to have elevated levels of glutathione (GSH). The MRP1-transfected line SKOV3-S2 was shown to be cross-resistant to doxorubicin, vincristine and etoposide but not to paclitaxel, vinblastine and platinum agents, such as cisplatin, JM216 [bis-acetato-ammine-dichloro-cyclohexylamine platinum (IV)] and AMD473 [*cis*-ammine dichloro (2-methyl-pyridine) platinum (II)]. No cross-resistance to any of the platinum agents was observed in a MRP1-overexpressing human lung cancer cell line with acquired doxorubicin resistance. Reduction of GSH levels (80–90%) by buthionine sulphoximine (BSO) produced significant potentiation in cisplatin sensitivity in the parental SKOV3, the vector-alone control SKOV3-puro and the MRP1-transfected line SKOV3-S2. The degree of sensitization was similar in all cell lines (1.6-fold). However, selective sensitization by BSO to vincristine was observed in the MRP1-transfected line (4.1-fold) but not in the vector control. No significant differences were observed in cisplatin accumulation in the SKOV3-puro and the SKOV3-S2 cells, although both these transfected lines accumulated significantly more than the parental line. Our results suggest that MRP1 does not play a significant role in platinum resistance in the human tumour cell lines investigated in this study.

**Keywords:** MRP1; platinum resistance; human ovarian carcinoma cell line

Multidrug resistance (MDR) has been shown to be caused by enhanced drug efflux mediated by two members of the ATP-binding cassette (ABC) family of transporter proteins, the 170-kDa P-glycoprotein (P-gp) (Juliano and Ling, 1976) and the 190-kDa multidrug resistance-associated protein (MRP1) (Cole et al, 1992). MRP1 was discovered because of its overexpression in a number of MDR human tumour cell lines that do not overexpress the P-gp.

Several reports have shown that MRP1 can act as a GS-X pump that is involved in the detoxification of heavy metals (Zaman et al, 1995; Ishikawa et al, 1996). This is mainly achieved by transporting drugs that are conjugated or co-transported with GSH (Muller et al, 1994; Jedlitschky et al, 1996). One of the main mechanisms of cisplatin resistance is increased cellular detoxification via elevated GSH (Andrews and Howell, 1990). Moreover, Ishikawa et al (1994) have shown overexpression of the GS-X pump in cisplatin-resistant human promyelocytic leukaemia HL-60 (HL-60/R-CP) cells, in which GSH was elevated. Conversely, other reports have suggested that MRP1 is not the major pump responsible for cisplatin resistance through studies in which the *MRP1* gene was transfected into a cervical cell line, HeLa (Cole et al, 1994), and into a non-small-cell lung cancer cell line (SW-1573) (Zaman et al, 1994), or using a pair of cisplatin-sensitive and acquired resistant sublines (De Vries et al, 1995).

*Received 25 September 1997*
*Revised 8 January 1998*
*Accepted 17 January 1998*

*Correspondence to:* SY Sharp, CRC Centre for Cancer Therapeutics, The Institute of Cancer Research, Block E, 15 Cotswold Road, Belmont, Sutton, Surrey, SM5 2NG, UK

Cisplatin is a widely used anti-cancer drug, particularly in the treatment of human ovarian, testicular, bladder, and head and neck cancers, but drug resistance is known to limit efficacy (Loehrer and Einhorn, 1984). In view of the above conflicting data, our aim in this study was to assess the possible role of MRP1 in platinum resistance primarily in human ovarian carcinoma cell lines, using established pairs of cisplatin-sensitive cells and cells with acquired cisplatin resistance, as well as parental lines of differing intrinsic sensitivity (>100-fold). Three pairs of lines with acquired cisplatin resistance have been studied; two in which transport defects have previously been described, 41M/41McisR (Loh et al, 1992) and A2780/A2780cisR (Holford et al, 1998), one in which elevated GSH has been shown to contribute to resistance, A2780/A2780cisR (Kelland et al, 1994) and the other (CH1/CH1cisR) in which resistance is attributable to DNA repair (Kelland et al, 1992). In addition, we have transfected a full-length MRP1 cDNA into an intrinsically cisplatin-resistant human ovarian carcinoma cell line, SKOV-3, and determined the drug resistance properties of the transfected line. This cell line was selected as it has been widely used in our previous studies of platinum drug resistance (Mistry et al, 1991; McKeage et al, 1994) and was shown to possess undetectable levels of MRP1 (see below). Our previous studies have shown that the main biochemical mechanisms of cisplatin resistance in the SKOV-3 cells are attributable to increased GSH levels (Mistry et al, 1991) and to the inhibition of formation of bifunctional interstrand DNA lesions, possibly by the formation of GSH adducts (McKeage et al, 1994). We have included investigation of two analogues of cisplatin of differing chemical reactivity and lipophilicity, JM216 (Kelland et al, 1993) and AMD473 (Holford et al, 1998), both recently introduced into the clinic.

Further platinum drug cross-resistance studies have been conducted using a pair of human non-small-cell lung cancer cell lines with acquired doxorubicin resistance, i.e. CORL23 and CORL23/R, in which the resistant line has been previously shown to overexpress MRP (Barrand et al, 1993).

### MATERIALS AND METHODS

Cisplatin and JM216 were synthesized by and obtained from the Johnson Matthey Technology Centre (Reading, Berkshire, UK). AMD473 was obtained from AnorMED (Langley, British Columbia, Canada). Chemicals were purchased from Sigma Chemicals (Poole, Hants, UK), unless otherwise stated.

### Cell lines

Five 'parent' human ovarian carcinoma cell lines were used in this study (SKOV3, 41M, CH1, A2780 and HX/62). Details of their establishment and biological properties have been described previously (Hills et al, 1989; Kelland et al, 1993). In addition, variants with acquired cisplatin resistance have been generated by continuously exposing cells to increasing concentrations of drug (41M/41McisR, CH1/CH1cisR, A2780/A2780cisR) (Behrens et al, 1987; Kelland et al, 1992). Cytotoxicity determinants for cisplatin against the panel of cell lines described above have been reported (Kelland et al, 1993). Briefly, the 96-h $IC_{50}$ values (μM) of cisplatin [as assessed by the sulphorhodamine B (SRB) assay] were 0.26 for 41M, 1.23 for 41McisR6 [resistance factor (RF) of 4.7], 0.11 for CH1, 0.71 for CH1cisR (RF of 6.4), 0.3 for A2780, 4.8 for A2780cisR (RF of 16), 4.4 for SKOV3 and 12.6 for HX/62 cell lines. The human non-small-cell lung cancer cell lines CORL23 and CORL23/R (doxorubicin resistant) were kindly provided by Dr P Twentyman (Cambridge, UK).

All cells grew as monolayers in Dulbecco's modified Eagle medium (DMEM) containing 10% heat inactivated fetal calf serum (Life Technologies, Scotland, UK), 2 mM L-glutamine, 0.5 μg $ml^{-1}$ hydrocortisone and minimal essential medium (MEM) non-essential amino acids in a 10% carbon dioxide, 90% air atmosphere, and all cell lines were free of Mycoplasma.

### Vector construction and transfection

The bicistronic plasmid vector pIRES-P (code number F250, EMBL accession number Z75185) was used to express MRP1 in eukaryotic cells. This vector expresses a single bicistronic mRNA driven from the human cytomegalovirus immediate early enhancer/promoter and uses the internal ribosome entry site from encephalomyocarditis virus to direct translation of the downstream *pac* gene encoding for puromycin acetyl transferase (de la Luna et al, 1988). Transfected clones surviving selection with puromycin are therefore biased towards expression of the upstream gene (Rees et al, 1996). A cDNA containing the complete coding sequence for MRP1 (Zaman et al, 1994) was cloned into the multiple cloning site of F250 as an *Nhe*I–*Not*I fragment, resulting in the vector F253 (Figure 1).

The intrinsically cisplatin-resistant cell line SKOV3 was transfected with the F250 vector or the vector containing the MRP1 coding sequence (F253 vector) using the cationic liposome-mediated transfection (lipofection) method (Boehringer Mannheim) and was performed according to the manufacturer's instructions. Briefly, $5 \times 10^5$ cells were seeded in each well of a six-well plate and were exposed to DNA (3 μg) and the transfection reagent (DOTAP) (8 μl) in serum-free Earle's balanced salt solution for 6 h. The content of the wells was then replaced with normal growth medium and incubated for a further 48 h. The cells were trypsinized and seeded into 24-well plates, and puromycin was added to the cells at 0.3 μg $ml^{-1}$ (a concentration previously determined to kill all non-transfected cells). Stable transfectants were selected after 3 weeks and analysed by Western blot. For this study, two transfected clones (SKOV3-S2 and SKOV3-S4) were used and were grown continuously for up to 6 months in normal growth medium (+ 10% FCS) in the absence of puromycin.

### Western blot

Cells ($1 \times 10^7$) were trypsinized, washed with phosphate-buffered saline (PBS) and suspended in 100 μl of lysis buffer [10 ml of 150 mM sodium chloride and 50 mM Tris-HCI, pH 7.5, 500 μl of phenylmethylsulphonyl fluoride (PMSF) (20 mM stock), 2 μl of Aprotinin (10 mg $ml^{-1}$ stock), 2 μl of Leupeptin (10 mg $ml^{-1}$ stock), 100 μl of sodium orthovanadate (10 mM stock), 100 μl of NP40 and 100 μl of 20% sodium dodecyl sulphate (SDS)] at 4°C for 1 h. Cells were then centrifuged at 12 000 r.p.m. (MSE Microcentrifuge) at 4°C for 15 min. The supernatant (total protein extract) was used for protein determination (Pierce BCA assay, Rockford, IL, USA) and Western blot analysis (Sharp et al, 1994). The mouse monoclonal antibody to MRP1 (MRP1m6) was kindly provided by Professor R Scheper, Amsterdam. The doxorubicin-resistant human large-cell lung cancer cell line COR-L23/R previously shown to overexpress MRP1 (Barrand et al, 1993) was used as the positive control.

### Assessment of cytotoxicity

Cytotoxicity (2 and 96 h) was measured by the SRB assay as described previously (Loh et al, 1992). The platinum agents, cisplatin (at 500 μM), JM216 (at 500 μM) and AMD473 (at 1 mM) were dissolved in 0.9% saline. Stock solutions of doxorubicin (Bristol Myers Squibb Pharmaceuticals), vinblastine (Farmitalia Carlo Erba, Milton Keynes, UK) and vincristine were made up to 1 mM and 500 μM, respectively, in sterile water. Etoposide (Farmitalia Carlo Erba) was dissolved at 34 mM, and paclitaxel (Bristol Myers Squibb Pharmaceuticals) was dissolved at 5 mM in ethanol. All aqueous drug solutions were filter sterilized before use.

The effect of GSH depletion by BSO on the cytotoxicity of cisplatin (and vincristine) was assessed in all cell lines according to Mistry et al (1991). Briefly, cells were exposed to 50 μM BSO (the highest non-toxic dose) or medium for 24 h. Different concentrations of cytotoxic drug were then added for 2 h, after which the drug was washed off and cell survival was assessed after 96 h as described above.

### GSH assay

The total GSH content of the cell lines was measured by an enzymatic assay using glutathione reductase (Mistry et al, 1991). The GSH content was expressed as nmol GSH per mg of protein. The effect of BSO on GSH levels was determined by treating cells ($1–4 \times 10^6$) with 50 μM BSO for 24 h, followed by GSH extraction.

### Intracellular platinum accumulation

The effect of concentration on cisplatin uptake (2 h) in the cell lines was determined as described previously using flameless

 © Cancer Research Campaign 1998

**Figure 1** The puromycin-resistant bicistronic IRES vector containing the complete coding sequence for MRP1 (F253 vector)

atomic absorption spectrometry (Perkin Elmer 1100B and HGA 700, Beaconsfield, Bucks, UK) (Loh et al, 1992). Cellular platinum levels were expressed as nmol platinum per mg of protein.

### RESULTS

### Western blot

The levels of expression of MRP1 in the panel of human ovarian cell lines and their variants with acquired cisplatin resistance were determined by immunoblotting using the MRP1m6 monoclonal antibody. The previously reported MRP1-positive doxorubicin-resistant human large-cell lung line (COR-L23/R) was included as a positive control. Figure 2A shows that there was no detectable expression of MRP1 in any of the cell lines.

MRP1 levels in the parental SKOV3, the vector-alone control SKOV3-puro and two selected MRP1-transfected clones (SKOV3-S2 and SKOV3-S4) were also determined. No over-expression of MRP1 was observed in the SKOV3 and SKOV3-puro lines (Figure 2B). The two MRP1-transfected clones overexpressed the 190-kDa MRP1, SKOV3-S2 having a higher expression of MRP1 than SKOV3-S4. The majority of subsequent experiments were performed using the SKOV3-S2 clone. There were no significant differences in doubling times between all cell lines (SKOV3, 23.1±4.0 h; SKOV3-puro, 19.3±2.3 h; and SKOV3-S2, 22.7±6.7 h).

**Figure 2** (**A**) Western blot for MRP1 in a panel of human ovarian cell lines. Lane 1, molecular weight markers; lane 2, COR-L23/R (positive control); lane 3, 41M; lane 4, 41McisR; lane 5, CH1; lane 6, CH1cisR; lane 7, A2780; lane 8, A2780cisR; lane 9, HX/62. (**B**) Western blot for MRP1 in the parental SKOV3 cells, the vector-alone control SKOV3-puro, and the two MRP1-transfected clones, SKOV3-S2 and SKOV3-S4

Further cross-resistance studies were conducted using the CORL23 and CORL23/R (MRP1 overexpressing) non-small-cell lung cancer lines (Table 1). The CORL23/R cell line was 23.5-fold resistant to doxorubicin and exhibited cross-resistance to etoposide and partial cross-resistance to vincristine and paclitaxel. However, non-cross-resistance, and even a trend of greater sensitivity, was observed to cisplatin, JM216 and AMD473.

### Cytotoxicity assessment

Cross-resistance profiles for SKOV3-puro and SKOV3-S2 to cisplatin, JM216, AMD473, doxorubicin, etoposide, paclitaxel and vinblastine showed that at 2- and 96-h drug exposure, the SKOV3-S2 line was cross-resistant to doxorubicin and etoposide only (Rf >1.5) (Figure 3). A similar cross-resistance profile was obtained in the SKOV3-S4 line, although the lower level of MRP1 expression did not correlate with a lower level of cross-resistance to doxorubicin (RF, 96 h exposure, 2.6) and etoposide (Rf 4.4). Rf values for the other drugs were: cisplatin 1.5, JM216 1.9, AMD473 1.6, paclitaxel 1.2 and vinblastine 1.3.

### Effect of BSO on cisplatin and vincristine sensitivity

To determine the effect of BSO on the GSH levels in SKOV3, SKOV3-puro and SKOV3-S2 cells, the total amount of GSH was determined. After 24-h exposure to 50 μM BSO, the levels of GSH were decreased by 80–90% in all cell lines (Table 2).

The 2-h cytotoxicity of cisplatin in the absence and presence of BSO was assessed in these cell lines. With the addition of BSO, there was a significant potentiation of cisplatin sensitivity in all cell lines. Reduction of GSH levels by BSO produced a similar degree of sensitization (1.6-fold) in the vector-alone control SKOV3-puro and the MRP1-transfected cell line SKOV3-S2 (Figure 4A). In

© Cancer Research Campaign 1998

**Figure 3** Cross-resistance profile of 2 h (open) and 96 h (shaded) of SKOV3-puro vs SKOV3-S2 to cisplatin, JM216, AMD473, doxorubicin, etoposide, paclitaxel and vinblastine. Resistance factor = $IC_{50}$ SKOV3-S2 line/$IC_{50}$SKOV3-puro line. Values are mean from ≥ three experiments

**Table 1** Cross-resistance profile of CORL23 vs CORL23/R (MRP positive)

| | CORL23 $IC_{50}$ (μM) | CORL23/R $IC_{50}$ (μM) | RF |
|---|---|---|---|
| Doxorubicin | 0.006 | 0.16 | 23.5 |
| | 0.026 | 0.52 | |
| Etoposide | 0.23 ± 0.046 | 12.2 ± 3.3 | 53 |
| Vincristine | 0.0158 ± 0.001 | 0.093 ± 0.008 | 5.9 |
| Paclitaxel | 0.0019 ± 0.00015 | 0.0066 ± 0.00025 | 3.5 |
| Cisplatin | 14 | 0.78 | 0.13 |
| | 12.5 | 2.5 | |
| JM216 | 3.4 | 0.63 | 0.32 |
| | 3.2 | 1.5 | |
| AMD473 | 32 | 5 | 0.29 |
| | 27 | 11 | |

Values represent mean ± s.d. ($n > 3$) or $n = 2$ (individual values given). RF, resistance factor ($IC_{50}$ L23R/L23 lines).

**Table 2** Intracellular GSH levels in the absence or presence of BSO in SKOV3, SKOV3-puro and SKOV3-S2 cell lines

| Cell line | Total GSH (nmol mg$^{-1}$ protein) | | Fold reduction (%) |
|---|---|---|---|
| | Without BSO | With BSO | |
| SKOV3 | 17.8±3.2 | 3.6±0.6 | 80 |
| SKOV3-puro | 14.8±2.2 | 2.2±0.6 | 85 |
| SKOV3-S2 | 21.3±5.7 | 2.2±0.6 | 90 |

Values indicate mean values ± s.d. $n \geq$ six experiments.

contrast, GSH depletion induced a selective potentiation of vincristine cytotoxicity in the MRP-overexpressing SKOV3-S2 cell line (4.1-fold) but not in the SKOV3-puro line (0.9-fold) (Figure 4B). The S2 MRP transfected subline was 7.8-fold resistant to vincristine compared with the SKOV3-puro control.

### Intracellular cisplatin accumulation

Figure 5 shows intracellular drug accumulation in the SKOV3, SKOV3-puro and SKOV3-S2 cells immediately after 2-h exposure to 10, 25 and 50 μM cisplatin. Across the range of concentrations, the vector-alone control SKOV3-puro accumulated

**Figure 4** (**A** and **B**) Cytotoxicity (2 h) of (**A**) cisplatin or (**B**) vincristine in the absence (open) or presence (shaded) of 50 μM BSO in the SKOV3, SKOV3-puro and SKOV3-S2 cells. The symbol * represents statistical significance ($P < 0.05$). Values for cisplatin are mean ± s.d. from ≥ four experiments

**Figure 5** Intracellular platinum accumulation in SKOV3 (■), SKOV3-puro (▲) and SKOV3-S2 (●) immediately after a 2-h exposure to 10, 25 and 50 μM cisplatin. Values are mean ± s.d. from three experiments significantly higher levels of cisplatin (3.6-fold) compared with the parental SKOV3 line. However, there were no significant differences in drug uptake between the SKOV3-puro and the MRP1-transfected clone SKOV3-S2.

### DISCUSSION

The MRP1 phenotype is characterized by resistance of cells to a range of MDR drugs, which include the anthracyclines and epidophyllotoxins, but, unlike P-glycoprotein-mediated resistance, it does not confer resistance to paclitaxel (Kavallaris, 1997). However, like P-glycoprotein, MRP1 is also related to reduced drug accumulation as a result of its function as an efflux pump.

© Cancer Research Campaign 1998

Studies have shown that MRP1 can act as a GS-X pump that is capable of transporting glutathione conjugates (Ishikawa et al, 1994; Muller et al, 1994; Jedlitschky et al, 1996). In addition, Zaman et al (1995), Schneider et al (1995) and Versantvoort et al (1995) have demonstrated that drug transport in MRP1- but not in P-glycoprotein-overexpressing cells can be regulated by manipulating intracellular GSH levels by BSO. As there are conflicting published data regarding the role of the MRP1/GS-X pump in determining platinum resistance (e.g. Fujii et al, 1994; Ishikawa et al, 1994, 1996; Muller et al, 1994; Zaman et al, 1994; De Vries et al, 1995), the present study is aimed at determining the role of MRP1 in platinum resistance in a panel of human ovarian carcinoma cell lines and their variants with acquired cisplatin resistance. Moreover, we have also transfected a MRP1 expression vector into an intrinsically cisplatin-resistant ovarian cell line, SKOV3, and investigated the platinum cross-resistant properties of the CORL23/CORL23R (doxorubicin-resistant) pair of lung cancer cell lines in which CORL23R is known to overexpress MRP1.

Our Western blot experiments have shown no overexpression of MRP1 in any of our panel of cisplatin-sensitive and -resistant ovarian cell lines. This appears to be in broad agreement with a study investigating MRP1 expression using immunohistochemistry across a wide panel of human cancer cell lines; in particular, ovarian lines tended to show relatively low expression (Izquierdo et al, 1996). Also, no detectable levels of P-glycoprotein were observed in any of the ovarian cell lines and in the SKOV3-puro and SKOV3-S2 lines (data not shown). The role of another newly described protein associated with multidrug resistance, termed the lung resistance protein (LRP or 110-kDa human vault protein) (Scheffer et al, 1995), was investigated in our panel of cisplatin-sensitive and -resistant cell lines. LRP overexpression has been found to predict a poor response to chemotherapy in acute myeloid leukaemia and ovarian carcinoma. However, no correlation between the expression of LRP and cisplatin sensitivity was observed in our cell line models, as detected by immunohistochemistry using the mouse monoclonal antibody LRP-56 (kindly provided by Professor RJ Scheper, Amsterdam) (data not shown). Transfection of MRP1 into the cell line SKOV3-S2 conferred resistance to doxorubicin, vincristine and etoposide but not to paclitaxel and vinblastine. These cross-resistance results are in agreement with observations made in a MRP1-transfected HeLa cell line (Cole et al, 1994) and the SW-1573 lung cancer cell line (Zaman et al, 1994).

Specifically in relation to platinum drugs, our data for cisplatin showing a lack of cross-resistance in the MRP1-transfected SKOV3 cell line are in agreement with those reported in the HeLa line (Cole et al, 1994). Our present work has extended the study of MRP1 and platinum drugs to include two compounds, the orally active, more lipophilic platinum IV drug JM216, now in phase II clinical trial, and AMD473, a sterically hindered platinum II agent shown to possess less reactivity toward GSH than cisplatin and about to enter a phase I clinical trial. For all three agents, there was no evidence of MRP1 contributing to resistance in the SKOV3 ovarian model. Moreover, there was a complete lack of cross-resistance observed to these platinum drugs in the CORL23/R (23-fold doxorubicin resistant, MRP1 positive) cell line, whereas resistance was observed to etoposide and vincristine. These results, combined with the lack of MRP1 expression across our panel of ovarian cell lines of differing intrinsic sensitivity to cisplatin (and varying levels of GSH) and in three sublines possessing acquired cisplatin resistance, suggest that MRP1 does not play a role in determining platinum drug sensitivity in these lines. While in agreement with the above reports using other MRP1-transfected lines, they differ from observations made by Ishikawa and colleagues using cisplatin-resistant murine leukaemia cells (Ishikawa et al, 1994, 1996). While the reasons for these differing findings are unknown, they may relate to cell line-specific effects (leukaemia vs ovarian carcinoma). The GS-X pump has been shown to play a role in the efflux of platinum in one other cisplatin-resistant human epidermoid cell line (KCP-4 cells, 25-fold resistant) (Fujii et al, 1994). In addition, another MRP1-related gene, the human canalicular multispecific organic anion transporter gene (*cMOAT* or *MRP2*) has recently been shown to be overexpressed in cisplatin-resistant human cancer cell lines with decreased drug accumulation (four to sixfold increase in mRNA expression) (Taniguchi et al, 1996).

As a further measure of possible importance of MRP1 in determining sensitivity to cisplatin, we have investigated the effects of manipulating GSH levels using the gamma glutamylcysteine synthetase inhibitor BSO, and we have measured cisplatin transport in the SKOV3 MRP1-transfected line. Many studies have reported a correlation between cisplatin resistance and increased intracellular detoxification via elevated GSH levels (for review, see Andrews and Howell, 1990). Our previous studies have shown that SKOV3 cells contain 2–4-fold higher GSH levels than the relatively cisplatin-sensitive cell lines 41M and CH1 (Mistry et al, 1991). The addition of BSO to SKOV3, the vector-alone control SKOV3-puro and the MRP1-transfected line SKOV3-S2 reduced the levels of GSH by 80–90%. However, resistance to cisplatin was significantly reversed (by depletion of GSH) in all cell lines regardless of MRP1 status (by 1.6-fold). In contrast, under identical experimental conditions, depletion of GSH by BSO selectively enhanced the cytotoxicity of vincristine 4.1-fold in the MRP1-transfected line, while no change in cytotoxicity was observed in the empty vector puromycin control line. Other studies have reported that the sensitivity of daunorubicin, vincristine and rhodamine 123 was enhanced in the presence of BSO in MRP1-overexpressing cell lines (Versantvoort et al, 1995). This effect of BSO on drug resistance was shown to be associated with an increase in intracellular drug accumulation. However, no significant differences in cisplatin uptake were observed between the SKOV3-puro and the SKOV3-S2 cells. Interestingly, there was a 3.6-fold increase in platinum accumulation in the SKOV3-puro and SKOV3-S2 lines compared with the parental SKOV3 line, although this was not manifest as a difference in $IC_{50}$ to cisplatin (2-h $IC_{50}$ values of SKOV3 and SKOV3-puro are 31.6±8.8 and 31.0±7.6 μM respectively). This difference in uptake could be attributable to the method of transfection (using cationic lipids), which may have an effect on the cell membrane of the transfected line or reflect clonal heterogeneity with the parental line.

In summary, taking together our data using human ovarian cell lines with intrinsic and acquired cisplatin resistance, MRP1-transfected lines and a doxorubicin-resistant MRP1-overexpressing line, there does not appear to be a significant role for MRP1 in platinum resistance in these models.

### ACKNOWLEDGEMENT

This work was supported by grants from the Cancer Research Campaign (UK).

© Cancer Research Campaign 1998 

### REFERENCES

Andrews PA and Howell SB (1990) Cellular pharmacology of cisplatin: perspectives on mechanisms of acquired resistance. *Cancer Cells* **2**: 93–100

Barrand MA, Rhodes T, Center MS and Twentyman PR (1993) Chemosensitisation and drug accumulation effects of cyclosporin A, PSC833 and verapamil in human MDR large cell lung cancer cells expressing a 190 K membrane protein distinct from P-glycoprotein. *Eur J Cancer* **29A**: 408–415

Behrens BC, Hamilton TC, Masuda H, Grotzinger KR, Whang-Peng J, Louie KG, Knutsen T, McKoy WM, Young R and Ozols RF (1987) Characterization of a *cis*-diamminedichloroplatinum (II)-resistant human ovarian carcinoma cell line and its use in evaluation of platinum analogs. *Cancer Res* **47**: 414–418

Cole SPC, Bhardwaj G, Gerlach JH, Mackie JE, Grant CE, Almquist KC, Stewart AJ, Kurz EU, Duncan AMV and Deeley RG (1992) Overexpression of a transporter gene in a multidrug-resistant human lung cancer cell line. *Science* **258**: 1650–1654

Cole SPC, Sparks KE, Fraser K, Loe DW, Grant CE, Wilson GM and Deeley RG (1994) Pharmacological characterization of multidrug resistant MRP1-transfected human tumor cells. *Cancer Res* **54**: 5902–5910

De Vries EGE, Muller M, Meijer C, Jansen PLM and Mulder NH (1995) Role of glutathione S-conjugate pump in cisplatin resistance. *J Natl Cancer Inst* **87**: 537–538

Fujii R, Mutoh M, Sumizawa T, Chen ZS, Yoshimura A and Akiyama S(1994) Adenosine triphosphate-dependent transport of leukotriene C4 by membrane vesicles prepared from cisplatin-resistant human epidermoid carcinoma tumor cells. *J Natl Cancer Inst* **86**: 1781–1784

Hills CA, Kelland LR, Abel G, Siracky J, Wilson AP and Harrap KR (1989) Biological properties of ten human ovarian carcinoma cell lines: calibration in vitro against four platinum complexes. *Br J Cancer* **59**: 527–534

Holford J, Sharp SY, Murrer BA, Abrams M and Kelland LR (1998) In vitro circumvention of cisplatin resistance by the novel sterically hindered platinum complex AMD473. *Br J Cancer* **77**: 366–373

Ishikawa T, Wright C and Ishizuka H (1994) GS-X pump is functionally overexpressed in *cis*-diamminedichloroplatinum (II)-resistant human leukemia HL-60 cells and down regulated by cell differentiation. *J Biol Chem* **269**: 29085–29093

Ishikawa T, Bao JJ, Yamane Y, Akimuru K, Frindrich K, Wright CD and Kuo MT (1996) Coordinated induction of MRP1/GS-X pump and gamma glutamylcysteine synthetase by heavy metals in human leukemia cells. *J Biol Chem* **271**: 14981–14988

Izquierdo MA, Shoemaker RH, Flens MC, Scheffer GL, Wu L, Prather TR and Scheper RJ (1996) Overlapping phenotypes of multidrug resistance among panels of human cancer cell lines. *Int J Cancer* **65**: 230–237

Jedlitschky G, Leier I, Buchholz U, Barnouin K, Kurz G and Keppler D (1996) Transport of glutathione, glucuronate, and sulfate conjugates by the *MRP1* gene-encoded conjugate export pump. *Cancer Res* **56**: 988–994

Juliano RL and Ling VA (1976) A surface glycoprotein modulating drug permeability in Chinese hamster ovary cell mutants. *Biochim Biophys Acta* **455**: 152–162

Kavallaris M (1997) The role of multidrug resistance-associated protein (MRP1) expression in multidrug resistance. *Anti-Cancer Drugs* **8**: 17–25

Kelland LR, Mistry P, Loh SY, O'Neill CF, Murrer BA and Harrap KR (1992) Mechanism-related circumvention of acquired *cis*-diamminedichloroplatinum (II) resistance using two pairs of human ovarian carcinoma cell lines by ammine/amine platinum (IV) dicarboxylates. *Cancer Res* **52**: 3857–3864

Kelland LR, Abel G, McKeage MJ, Jones M, Goddard PM, Valenti M, Murrer BA and Harrap KR (1993) Preclinical antitumor evaluation of bis-acetato-ammine-dichloro-cyclohexylamine platinum (IV): an orally active platinum drug. *Cancer Res* **53**: 2581–2586

Kelland LR, Barnard CFJ, Mellish KJ, Jones M, Goddard PM, Valenti M, Bryant A, Murrer BA and Harrap KR (1994) A novel *trans*-platinum coordination complex possessing *in vitro* and *in vivo* antitumor activity. *Cancer Res* **54**: 5618–5622

Loehrer PJ and Einhorn LH (1984) Cisplatin. *Ann Int Med* **100**: 704–713

Loh SY, Mistry P, Kelland LR, Abel G and Harrap KR (1992) Reduced drug accumulation as a major mechanism of acquired resistance to cisplatin in a human ovarian carcinoma cell line: circumvention studies using novel platinum (II) and (IV) ammine/amine complexes. *Br J Cancer* **66**: 1109–1115

de la Luna S, Soria I, Pulido D, Ortin J and Jimenez A (1988) Efficient transformation of mammalian cells with constructs containing a puromycin-resistance marker. *Gene* **62**: 121–126

McKeage MJ, Abel G, Kelland LR and Harrap KR (1994) Mechanism of action of an orally administered platinum complex [ammine bis butyrato cyclohexylamine dichloroplatinum (IV) (JM221)] in intrinsically cisplatin-resistant human ovarian carcinoma *in vitro*. *Br J Cancer* **69**: 1–7

Mistry P, Kelland LR, Abel G, Sidhar S and Harrap KR (1991) The relationships between glutathione, glutathione-S-transferase and cytotoxicity of platinum drugs and melphalan in eight human ovarian carcinoma cell lines. *Br J Cancer* **64**: 215–220

Muller M, Meijer C, Zaman GJR, Borst P, Scheper RJ, Mulder NH, de Vries EGE and Jansen PLM (1994) Overexpression of the gene encoding the multidrug resistance-associated protein results in increased ATP-dependent glutathione S-conjugate transport. *Proc Natl Acad Sci USA* **91**: 13033–13037

Rees S, Coote J, Stables J, Goodson S, Harris S and Lee MG (1996) Bicistronic vector for the creation of stable mammalian cell lines that predisposes all antibiotic-resistant cells to express recombinant protein. *Bio Techniques* **20**: 102–110

Scheffer GL, Wijngaard PLJ, Flens MJ, Izquierdo MA, Slovak ML, Pinedo HM, Meijer CJLM, Clevers HC and Scheper RJ (1995) The drug resistance-related protein LRP is the human major vault protein. *Nature Med* **1**: 578–582

Schneider E, Yamazaki H, Sinha BK and Cowan KH (1995) Buthionine sulphoximine-mediated sensitisation of etoposide-resistant human breast cancer MCF7 cells overexpressing the multidrug resistance-associated protein involves increased drug accumulation. *Br J Cancer* **71**: 738–743

Sharp SY, Rowlands MG, Jarman M and Kelland LR (1994) Effects of a new antioestrogen, idoxifene, on cisplatin- and doxorubicin-sensitive and -resistant human ovarian carcinoma cell lines. *Br J Cancer* **70**: 409–414

Taniguchi K, Wada M, Kohno K, Nakamura T, Kawabe T, Kawakami M, Kagotani K, Okumura K, Akiyama SI and Kuwano M (1996) A human canalicular multispecific organic anion transporter (cMOAT) gene is overexpressed in cisplatin-resistant human cancer cell lines with decreased drug accumulation. *Cancer Res* **56**: 4124–4129

Versantvoort CHM, Broxterman HJ, Bagrij, Scheper RJ and Twentyman PR (1995) Regulation by glutathione of drug transport in multidrug-resistant human lung tumour cell lines overexpressing multidrug resistance-associated protein. *Br J Cancer* **72**: 82–89

Zaman GJR, Flens MJ, van Leusden MR, de Haas M, Mulder HS, Lankelma J, Pinedo HM, Scheper RJ, Baas F, Broxterman HJ and Borst P (1994) The human multidrug resistance-associated protein MRP1 is a plasma membrane drug-efflux pump. *Proc Natl Acad Sci USA* **91**: 8822–8826

Zaman GJR, Lankelma J, van Tellingen O, Beijnen J, Dekker H, Paulusma CC, Oude Elferink RP, Baas F and Borst P (1995) Role of glutathione in the export of compounds from cells by the multidrug resistance-associated protein. *Proc Natl Acad Sci USA* **92**: 7690–7694

*© Cancer Research Campaign 1998*